# Mesenchymal stem cells for the treatment of ulcerative colitis: a systematic review and meta-analysis of experimental and clinical studies

**DOI:** 10.1186/s13287-019-1336-4

**Published:** 2019-08-23

**Authors:** Xiao Shi, Qi Chen, Fen Wang

**Affiliations:** 1 Department of Gastroenterology, The Third Xiangya Hospital, Central South University, Changsha, 410013 Hunan People’s Republic of China; 2 Department of Dermatology, Xiangya Hospital, Central South University, Changsha, 410008 Hunan People’s Republic of China; 3 Department of Gastroenterology, Hunan Key Laboratory of Non-Resolving Inflammation and Cancer, The Third Xiangya Hospital, Central South University, 138 Tongzi Road, Changsha, 410013 Hunan People’s Republic of China

**Keywords:** Mesenchymal stem cells, Ulcerative colitis, Systematic review and meta-analysis, Animal studies, Clinical trials, Disease activity index, Histopathological score, Colon length, Healing rate

## Abstract

**Objective:**

To explore the promising use of mesenchymal stem cells (MSCs) for ulcerative colitis (UC).

**Methods:**

Studies reporting MSC treatment on UC were searched on five databases. Methodological quality was assessed based on the SYRCLE’s Risk of Bias (RoB) tool and MINORS tool. Data analysis was conducted using Engauge Digitizer 10.8 and Stata 14.0.

**Results:**

A total of 15 studies met the inclusion criteria including 8 animal (*n* = 132) and 7 human (*n* = 216) trials. In animal studies, mice treated with MSCs had significantly lower disease activity index (DAI) than that in the control group: the 1st day (standardized mean difference (SMD) − 0.753, *p* = 0.027), the 3rd day (SMD − 1.634, *p* = 0.000), the 5th day (SMD − 2.124, *p* = 0.000), the 7th day (SMD − 5.327, *p* = 0.000), the 9th day (SMD − 2.979, *p* = 0.000), and the 14th day (SMD − 5.032, *p* = 0.000). Lower histopathological score (HS) (SMD − 5.15, *p* < 0.05) and longer colon length (SMD 2.147, *p* = 0.001) in mice treated with MSCs were also indicated. The main outcome in clinical trials showed, compared with control group, healing rate of patients accompanied by MSC therapy elevated obviously: MSCs vs 5-aminosalicylic acids (5-ASA) (RR = 2.317, *p* = 0.000) and MSCs + 5-ASA vs placebo + 5-ASA (RR = 5.118). The analytical data in 4 trials conducted with single-arm studies also demonstrated increased healing rate (0.787) after MSC treatment (*p* = 0.000).

**Conclusion:**

Our meta-analysis results supported that MSCs could be an underlying method of treating UC.

**Electronic supplementary material:**

The online version of this article (10.1186/s13287-019-1336-4) contains supplementary material, which is available to authorized users.

## Introduction

Ulcerative colitis (UC) is a chronic, idiopathic inflammation of the large intestine (colon), which is classified as a form of inflammatory bowel disease (IBD) [1]. It is characterized by suffering from a relapsing and remitting course [2]. Both male and female are affected equally, specially adults aged 30–40 years [3]. The incidence of UC has been increasing around the world. The highest annual incidence reported was 24.3 per 100,000 person-years in Europe, 6.3 per 100,000 person-years in Asia and the Middle East, and 19.2 per 100,000 person-years in North America [4]. In patients with UC, ulcers and inflammation of the inner lining of the colon could incur symptoms of abdominal pain, diarrhea, and rectal bleeding [5]. The exact cause of UC remains unknown. Current studies have shown that abnormal activation of the immune system, hereditary susceptibility and alteration of intestinal flora caused by mucosal barrier defects may play a role in the pathophysiology of UC [6–8].

The existing clinical managements include conventional medications, endoscope therapy, and surgery treatment. Majority of UC patients would be subject to medications including anti-inflammatory agents such as 5-aminosalicylic acids (5-ASA), systemic corticosteroids, and topical corticosteroids, as well as immunomodulators like azathioprine, 6-mercaptopurine (6-MP), cyclosporine, and methotrexate [9]. Unfortunately, it is difficult to cure UC completely, with 74% of patients experiencing at least one relapse during 5-year observation in a prospective population-based cohort study [10]. A meta-analysis conducted by Ford et al. [11] has shown that 887 (60.3%) of 1470 UC patients fell short of achieving remission in randomized to receive 5-ASA, indicating that more than half of UC patients may not be able to have a positive response to traditional medications. What is more, taking these drugs could lead to the occurrence of various adverse effects [12]. The use of corticosteroids is confirmed to be associated with cutaneous effects, weight gain, hyperglycemia, osteoporosis, adrenal insufficiency, and cataracts [13]. Moreover, corticosteroid therapy is capable of increasing risk of opportunistic infections, especially when administered in combination with other immunosuppressive drugs [14]. The intolerance or potential occurrence of myelotoxicity and hepatotoxicity generated by immunomodulators could make nearly one fourth of patients discontinue the treatments [15, 16]. Therefore, new therapeutic targets are required in order to achieve ameliorative efficacy without a risk of incontinence.

Mesenchymal stem cells (MSCs) are one of the most popular multipotent stem cells which have been widely explored over the past few decades [17]. MSCs have shown therapeutic effects in various inflammatory diseases and kidney transplantation due to its hypo-immunogenic and immunoregulatory properties [18–22]. MSCs could be easily isolated and amplified from the bone marrow and other tissues [23, 24]. Previous reviews have demonstrated that MSCs could regulate innate and adaptive immune responses by releasing various mediators, including immunosuppressive molecules, growth factors, exosomes, chemokines, complement components, and multiple metabolites, when exposed to inflammatory environment, thus promoting the repair and regeneration of damaged tissues [25].

The first animal experiment to investigate MSCs for treatment of UC mouse model was conducted in 2006. The results showed that bone marrow-derived MSCs played a role in repairing injured intestinal mucosa, as well as downregulating the immune function of T cells [26]. In 2009, the successful application of MSCs in UC patients was reported for the first time [27]. However, there are scarce large-scale prospective trials that could convincingly evaluate the efficiency and safety of MSC as a candidate therapeutic strategy for UC. As such, the objective of our study was to perform a systematic review and meta-analysis of animal and clinical studies on the treatment of UC with MSCs.

## Material and methods

### Search strategy

A comprehensive search was performed in electronic database as follows: PubMed, EMBASE, the Cochrane Library of Systematic Reviews, Web of Science, and China National Knowledge Infrastructure. Free text words and database-specific index terms were combined with Boolean operators (“ AND “ and “ OR “) to improve the sensitivity of our search. The identified studies were not constrained by publication date, language, or publication status. The following search strategy was applied: (Mesenchymal stem cells, Bone Marrow Stromal Cells, Mesenchymal Progenitor Cells, Mesenchymal Stromal Cells) AND (Ulcerative Colitis, Idiopathic Proctocolitis, Colitis Gravis). Retrieval strategy is shown in Additional file 1.

### Study selection

All study selections were conducted by two reviewers (Xiao Shi and Qi Chen) independently, with discrepancies discussed with the research group. We applied the following inclusion criteria: (1) published or unpublished single-arm studies, randomized controlled trial (RCT), or non-RCT with or without full texts; (2) included patients with UC; (3) animal trials with or without full texts; and (4) MSCs as a therapy for the treatment of UC without restricting the type of MSC, dose of cells, and the route of MSC administration. Exclusion criteria were as follows: (1) repeated studies, (2) no original research (reviews, editorials, non-research letters, protocols), (3) no separation of UC and Crohn’s disease (CD), and (4) observational studies. Foreign language articles were translated by professional translation software when necessary. Articles of meetings were manually searched to ensure that they were published only in abstract forms.

### Data extraction

Two independent authors (Xiao Shi and Qi Chen) evaluated titles and abstracts and resolved conflicts through discussion and consensus. Full texts were screened to extract all of the data from each eligible study. On the part of experiments in mice, the data contained the following: (1) first author; (2) year; (3) location; (4) mouse sex, strain, and weight; (5) number of each group; (6) modeling method; (7) modeling duration; (8) type and source of MSCs; (9) way of MSCs administrated; (10) times of treatment; and (11) parameter. For clinical trials, the data contained the following: (1) first author, (2) year, (3) location, (4) type of study, (5) number of MSCs group, (6) number of control group, (7) male/female, (8) age, (9) type and source of MSCs, (10) way of MSCs administrated, (11) outcomes, and (12) adverse events.

### Assessment of study quality and bias

Varying quality assessment tools were used to evaluate the bias risk of each enrolled study.

In terms of animal experiments, six parts including the title, abstract, introduction, methods, results, and discussion were explored using the SYRCLE’s risk of bias (RoB) tool where the criteria contained 6 sorts of bias with 10 items. Each item contains several details and was classified as low, unclear, and high risk of bias [28]. The (MINORS) tool, involving 8 and 12 items for clinical trials with and without control groups respectively, was adopted to assess the quality of included clinical trials [29].

### Statistical analysis

Disease activity index (DAI) was a potential factor to reflect the severity of UC, which involved the assessment of the character of stool and occult blood [30]. The morphological and pathological changes of UC could be represented by the evaluation of colon length and histopathological score (HS). Therefore, standardized mean difference (SMDs) and related 95% CIs of DAI, colon length as well as HS in both treatment and control groups were retrieved in animal studies. For each human study, the outcome of healing rate (HR) was considered as the main endpoint. Odds ratios (ORs) and related 95% CIs were calculated to compare treatment with control groups. For each eligible study, if the associated information was present merely in figures, two reviewers (Xiao Shi and Qi Chen) would use Engauge Digitizer 10.8 to collect data from the statistical graphs independently. Then, the mean values would be adopted [31]. For animal studies, there always existed huge differences in modeling duration and time point of intervention between different trials. In order to obtain comparability, the day of intervention was defined as the first day of data recording.

We evaluated the degree of heterogeneity between studies using inconsistency index (*I*^2^). Values of *I*^2^ equal to 25, 50, and 75% were considered to indicate low, moderate, and high heterogeneity, respectively [32]. If *I*^2^ < 50%, a fixed-effects model was applied; otherwise, a random-effects model was used [33]. With the purpose of exploring the sources of heterogeneity, all of the enrolled studies were sequentially excluded to demonstrate the overall impact of individual study and performed with subset analysis of time and treatment intervention afterwards where *I*^2^ > 50%. Statistical meta-analysis was performed in STATA version 14.0 to generate forest plots of pooled ORs and SMDs with 95% CIs.

## Results

### Search results

**Fig. 1:**
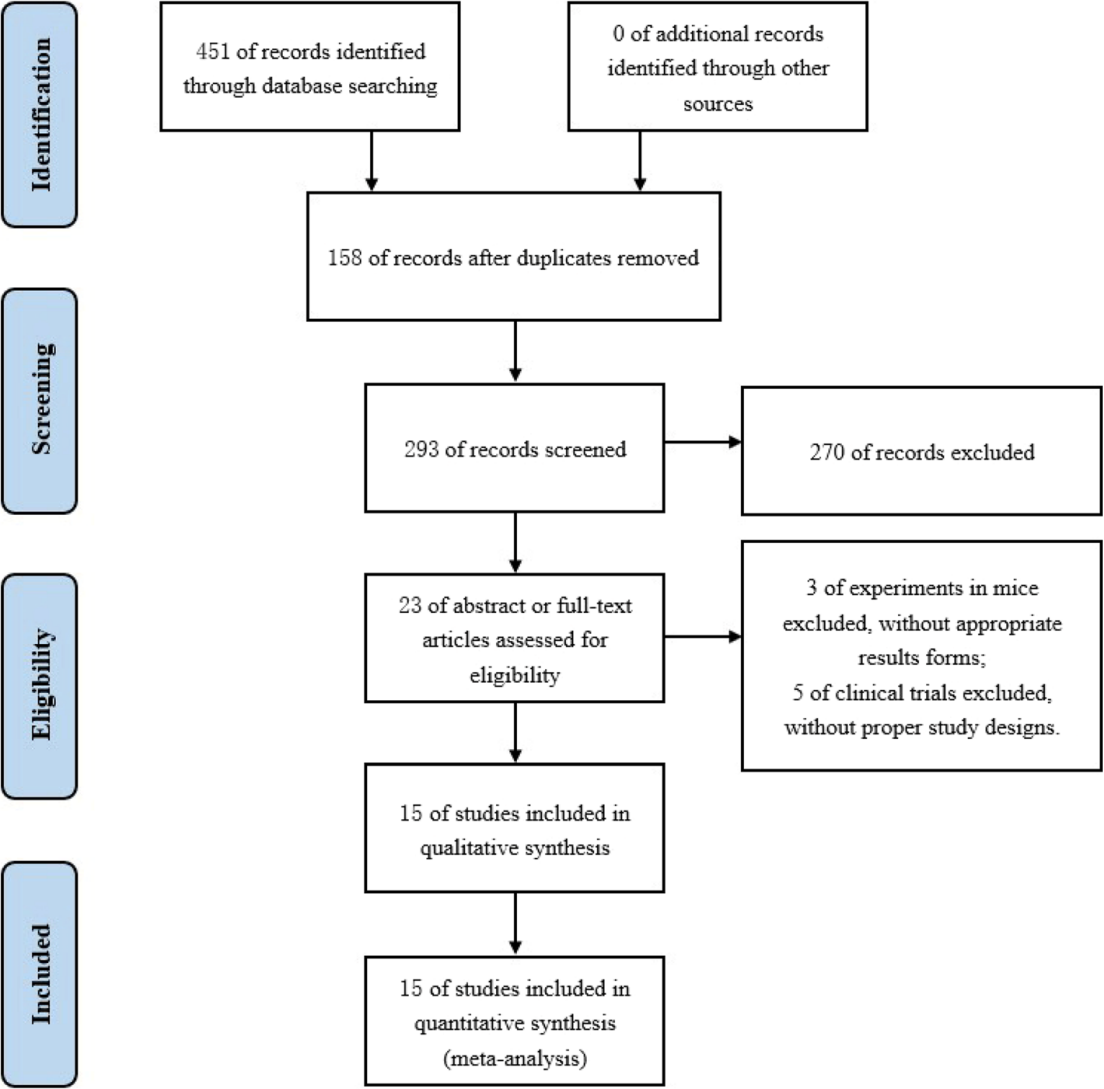
Flow chart showing the meta-analysis study selection

A total of 451 references were identified for review, of which 158 were excluded due to duplication. After reading through titles and abstracts, 270 studies were excluded for being irrelevant. Twenty-three possible full-text studies were carefully reviewed. Three clinical studies were excluded because of inappropriate result forms, and another 5 animal studies were excluded resulting from improper study designs. Ultimately, 7 human [34–40] and 8 animal studies [41–48] were selected for the meta-analysis (Fig. 1).

### Animal studies

#### Study characteristics

A total of 132 mice were reported. C57BL mice made up 63.6% of the total number of mice used; BALB/C mice accounted for 36.4%. Male mice accounted for 84.8%, and female mice for the remaining 15.2%. All of the 8 studies applied the same modeling method: UC mouse model was induced by receiving dextran sodium sulfate (DSS) drinking water instead of regular drinking water in control groups. Study characteristics are shown in Table 1.

**Table 1:** Characteristics of mouse experiments

First author	Year	Location	Mice (sex, strain, weight)	Number of each group	Modeling method	Modeling duration	Type and source of MSCs	Way of MSCs administrated	Times of treatment	Parameter
Guo-Chao Niu	2012	China	Male, C57B L/6, 18~22 g	DSS + Vechile (*n* = 10) DSS + MSCs (*n* = 10)	DSS (5%)	30	Mouse UC-MSCs	Tail vein injection	1	DAI, colon length, HS
Xiao-Wen He	2012	China	Male, BALB/c mice, 19–21 g	DSS + 4%PBS (*n* = 6) DSS + MSCs (*n* = 6)	DSS (4%)	7	Mouse BM-MSCs	Tail vein injection	3	DAI, colon length, HS
Xiao-Xi Xu	2018	China	Male, BALB/c mice, 18–22 g	DSS + 4%PBS (*n* = 8) DSS + ERCs (*n* = 8)	DSS (3%)	7	Mouse ERCs	Tail vein injection	3	DAI, colon length
Jin Seok Park	2015	Korea	Male, C57BL/6 mice, N/A	DSS + PBS (*n* = 8) DSS + mc-MSCs (*n* = 8)	DSS (2.5%)	6	MC-MSCs	Tail vein injection	3	DAI, colon length, HS
Young-Sun Nam	2015	South Korea	Female, C57BL/6, N/A	DSS + PBS (*n* = 10) DSS + MSCs (*n* = 10)	DSS (3.5%)	6	Mouse BM-MSCs	Intraperitoneal injection	1	DAI, colon length
Wei-Xin Liu	2015	China	Male, C57BL/6 mice, N/A	DSS (*n* = 10) DSS + MSCs (*n* = 10)	DSS (N/A)	7	Mouse BM-MSCs	Tail vein injection	1	DAI
Forte	2015	Italy	Male, C57BL/6 mice, N/A	DSS (*n* = 4) DSS + MSCs (*n* = 4)	DSS (1.5%)	9	Human AD-MSCs	Irrigation	3	DAI, HS
Li Cao	2019	China	Male, BALB/c mice, 21–23 g	DSS + NS (*n* = 10) DSS + EVs (*n* = 10)	DSS (3%)	7	EVs from Mouse BM-MSCs	Intraperitoneal injection	7	DAI, colon length

*DSS* dextran sodium sulfate, *MSCs* mesenchymal stem cells, *PBS* phosphate buffer saline, *NS* normal saline, *EVs* extracellular vesicles, *ERCs* endometrial regenerative cells, *MC-MSCs* mouse clonal MSCs, *UC-MSCs* umbilical cord MSCs, *BM-MSCs* bone marrow MSCs, *AD-MSCs* adipose-derived MSCs, *DAI* disease activity index, *HS* histopathological score

#### Quality of studies

According to the SYRCLE’s RoB tool, all of the animal studies were moderate to high for risk of bias. The SYRCLE risk of bias assessment revealed a low risk of 40%, unclear risk of 21.3%, and high risk of 38.7% among them.

Only 3 in 8 studies mentioned random sequence generation. It was hard to confirm the accurate baseline characteristics of mice in each group because none of the studies offered completed baseline information. It seemed that there is a lack of standard practice for allocation concealment and blinding of both study personnel and outcome assessors in all 8 studies. No study described any blindness so that both performance and detection bias were high. Attrition and reporting bias were low because outcomes in all 8 studies were clear and sufficient. The details can be found in Table 2.

**Table 2:**
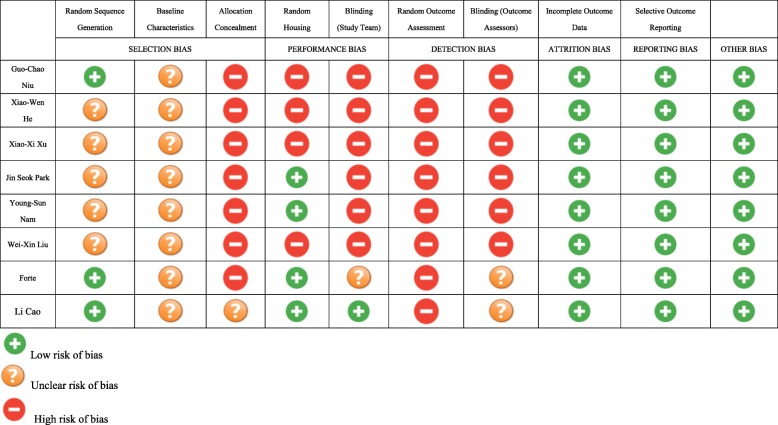
SYRCLE’s RoB tool for each experimental animal studies

#### DAI

**Fig. 2:**
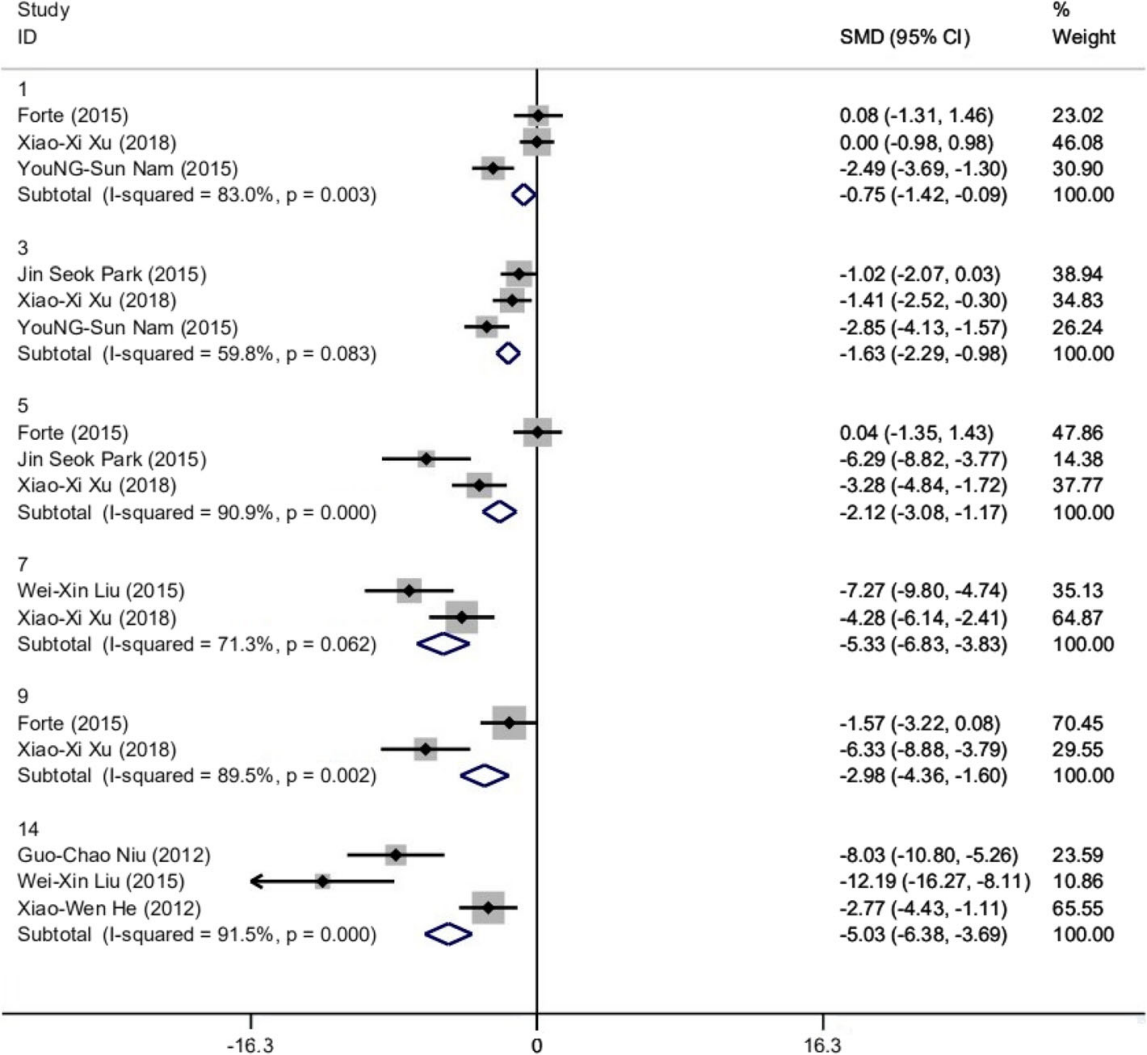
Forest plot of mouse experiments about DAI

All of the 8 studies reported DAI; however, the data from Cao was excluded on account of the DAI was measured with mean level (0 days to 7 days), which was lack of comparability. We divided the time points of DAI assessment into six subgroups: 3 (*n* = 44), 3 (*n* = 52), 3 (*n* = 40), 2 (*n* = 36), 2 (*n* = 24), and 3 (*n* = 52) studies belong to the 1st day, the 3rd day, the 5th day, the 7th day, the 9th day, and the 14th day, respectively. The random-effects model and Cohen’s method were used to assess the differences in DAI between the treatment group and control group. Subgroup results showed that the level of DAI was lower in the treatment group and there were significant differences between the two groups: the 1st day (SMD − 0.753, 95% CI − 1.418 to − 0.088, *p* = 0.027; *I*^2^ = 83.0%, *p* = 0.003), the 3rd day (SMD − 1.634, 95% CI − 2.289 to − 0.979, *p* = 0.000; *I*^2^ = 59.8%, *p* = 0.083), the 5th day (SMD − 2.124, 95% CI − 3.083 to − 1.165, *p* = 0.000; *I*^2^ = 90.9%, *p* = 0.000), the 7th day (SMD − 5.327, 95% CI − 6.827 to − 3.827, *p* = 0.000; *I*^2^ = 71.3%, *p* = 0.062), the 9th day (SMD − 2.979, 95% CI − 4.361 to − 1.597, *p* = 0.000; *I*^2^ = 89.5%, *p* = 0.002), and the 14th day (SMD − 5.032, 95% CI − 6.376 to − 3.689, *p* = 0.000; *I*^2^ = 91.5%, *p* = 0.000) (Fig. 2). Studies were heterogeneous in each subgroup. To explore the sources of heterogeneity, linear regression was conducted, which suggested that the subgroup analysis could explain the heterogeneity by 44.83% (Additional file 2: Table S1).

#### Colon length

**Fig. 3:**
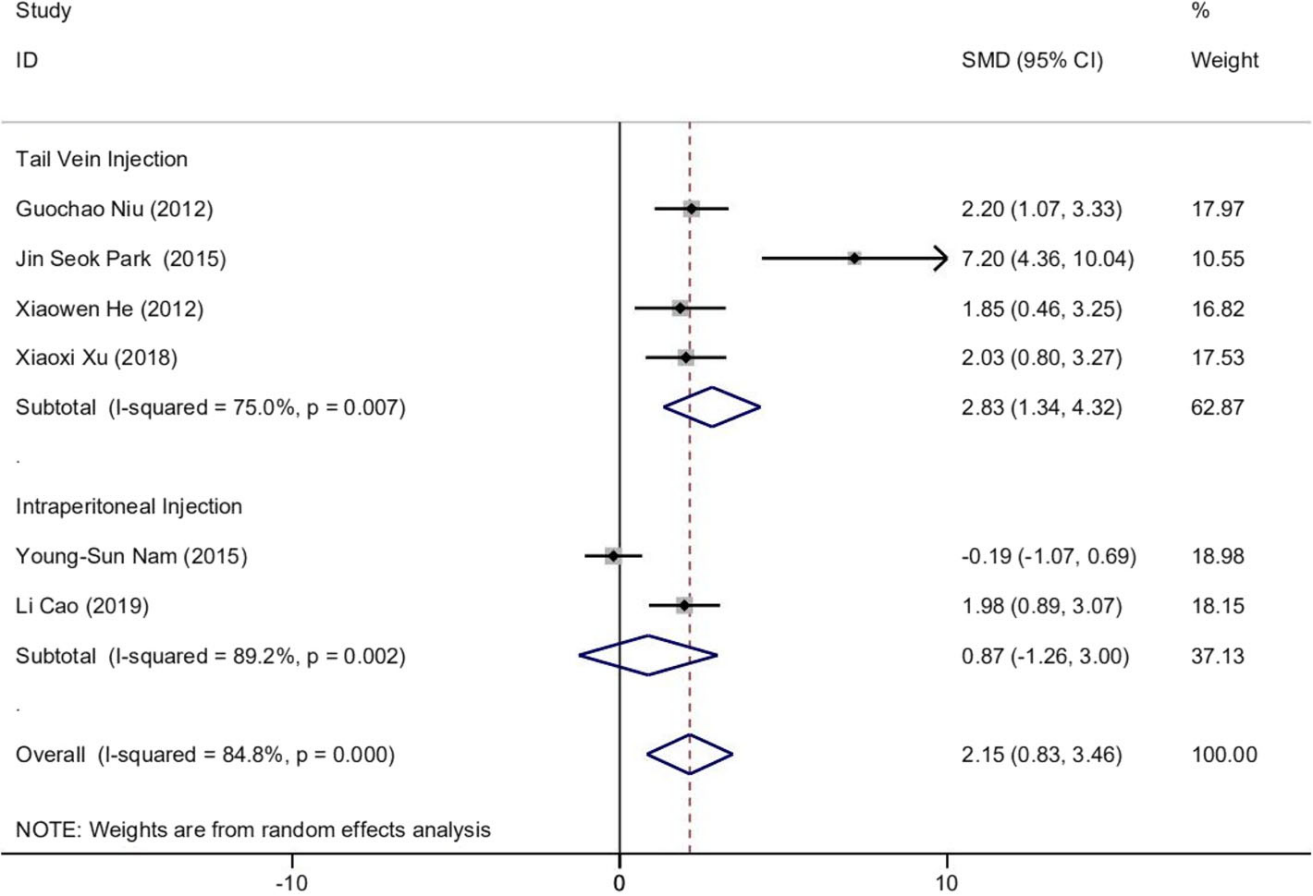
Forest plot of mouse experiments on colon length

Six of the 8 studies reported colon length (*n* = 104). We applied the random-effects model and Cohen’s method to evaluate the differences in colon length between the treatment group and control group. The MSC experimental group demonstrated a clear increase in colon length compared to the control group (SMD 2.147, 95% CI 0.830 to 3.463, *p* = 0.001; *I*^2^ = 84.8%, *p* = 0.000) (Fig. 3). Additionally, subgroup analysis based on administration routes was carried on. It was indicated that tail vein injection has a more stable outcome (SMD 2.830, 95% CI 1.343 to 4.316, *p* = 0.000; *I*^2^ = 75.0%, *p* = 0.007) than intraperitoneal injection (SMD 0.871, 95% CI − 1.258 to 3.001, *p* = 0.423; *I*^2^ = 89.2%, *p* = 0.002) (Fig. 3). To explore the sources of heterogeneity, sensitivity analysis was performed by excluding studies sequentially. The results showed that after excluding the study by Park et al. [37] and Nam et al. [38], the heterogeneity decreased to low level (*I*^2^ = 0.000, *p* = 98.5%) (Additional file 2: Table S2).

#### Histopathological score

**Fig. 4:**
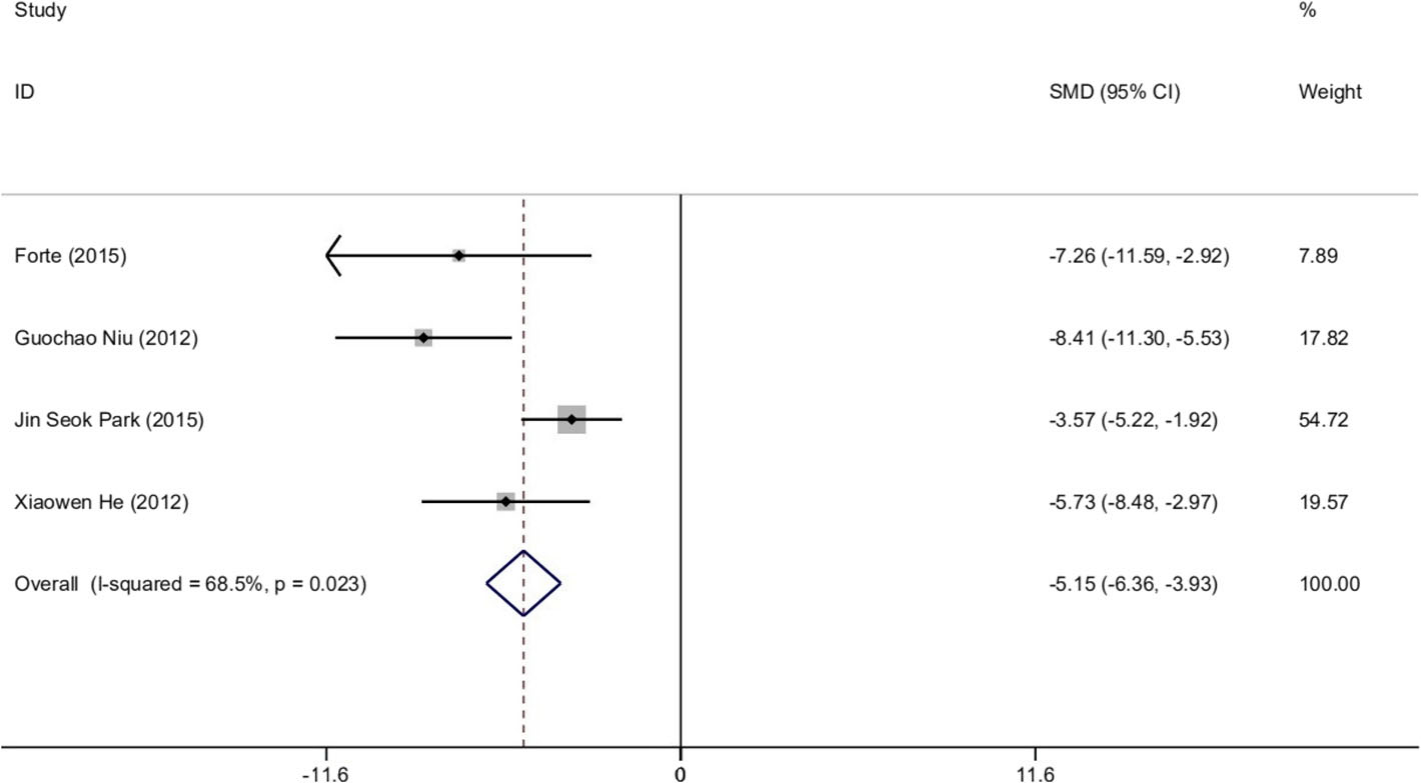
Forest plot of mouse experiments about histopathological score

Four of the 8 studies reported HS (*n* = 56). The random-effects model and Cohen’s method was applied to evaluate the differences in histopathological score between the treatment group and control group. The MSC experimental group cleared a significant decrease in HS compared to the control group (SMD − 5.15, 95% CI − 1.16 to 0.53, *p* < 0.05; *I*^2^ = 68.5%, *p* = 0.023) (Fig. 4). To explore the sources of heterogeneity, the studies were excluded in sequence. We noticed that by excluding the study conducted by Park et al. [37], the heterogeneity decreased to moderate level (*I*^2^ = 41.5%), which suggested the main source of the heterogeneity (Additional file 2: Table S3).

### Human studies

#### Description of studies

A total of 216 patients were included. Of them, 139 received intravenous infusions, 33 adopted submucous injections through colonoscopy, and the remaining 44 were unclear. Study demographics and clinical characteristics are summarized in Table 3. Four of these studies were single-arm clinical trials, two were non-RCTs, and one was RCT. Remarkably, no serious adverse events were reported.

**Table 3:** Characteristics of clinical trials

First author	Year	Location	Type of study	Number of MSC group	Number of control group	Male/female	Age	Type and source of MSCs	Way of MSCs administrated	Outcomes	Adverse events	MINORS
Knyazev, O.	2017	Russia	Meeting abstract	26	N/A	N/A	20–62 (mean 28)	BM-MSCs	Submucosal injection by colonoscopy	One-year healing rate 23/26	N/A	9
Yang, Bo.	2015	China	Full text	7	10	13/4	37–62	BM-MSCs	Submucosal injection by colonoscopy	14-month healing rate 7/7, 3/10	N/A	16
Lazebnik, L.	2011	Russian	Meeting abstract	44	N/A	N/A	N/A	BM-MSC	N/A	One-year healing rate 32/44	N/A	9
Jun Liang	2012	China	Letter	3	N/A	1/2	22–44 (mean 29)	BM-MSC	N/A	One-year healing rate 2/3	N/A Insomnia; low; fever	7
Lazebnik, L.	2010	Russian	Meeting abstract	44	N/A	N/A	N/A	BM-MSC	Intravenous Infusions	Two-year healing rate 34/44	N/A	9
Knyazev, Oleg	2013	Russian	Meeting abstract	58	50	N/A	19–64 (mean 36)	BM-MSC	Intravenous Infusions	One-year healing rate 44/58, 17/50	N/A	16
Jian-Xia Hu	2016	China	Full text	34	36	21/13 22/14	42.9 ± 23.1 and 43.7 ± 28.7	UC-MSCs	Intravenous infusions	One-year healing rate 85.3%, 16.7%	N/A	22

*BM-MSCs* bone marrow MSCs, *UC-MSCs* umbilical cord MSCs, *MINORS* methodological index for non-randomized studies

#### Quality of studies

The qualities of studies included in our analysis are shown in Table 3. Four studies are single-arm clinical trials with a maximum score of 16 points while the other 3 studies with control groups get a maximum score of 24 points. Only one study got access to high scores (22 points), while the others did not. It was the lack of inclusion of consecutive patients, unbiased assessment of the study endpoint, and prospective calculation of sample size that might be attributed to. In total, the quality of clinical trials is poor.

#### Clinical trials without the control group

**Fig. 5:**
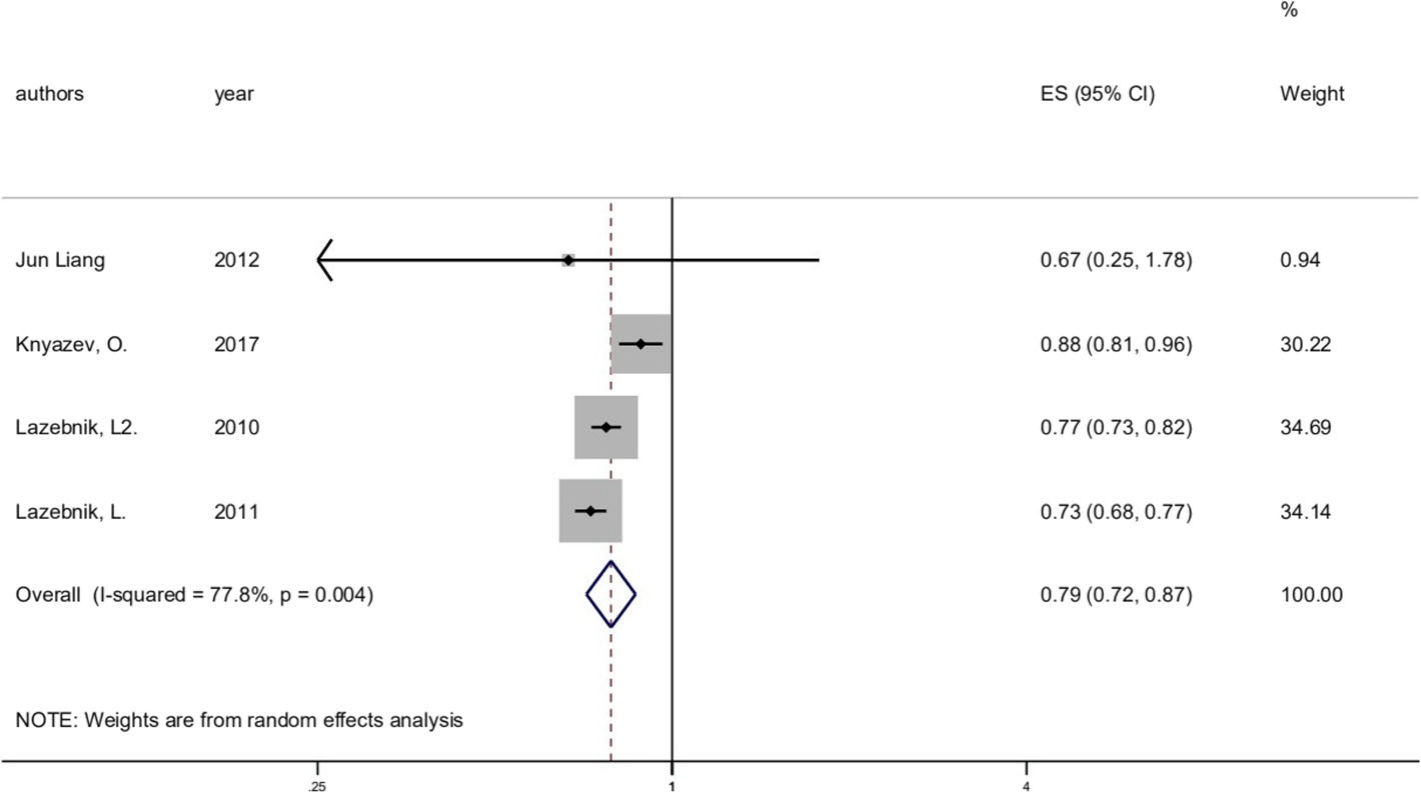
Forest plot of clinical trials without the control group

For 4 articles involved, the overall healing rate was 0.787 (95% CI 0.715 to 0.867, *p* = 0.000; *I*^2^ = 77.8%, *p* = 0.004) among 117 patients with UC (Fig. 5).

#### Clinical trials with the control group

**Fig. 6:**
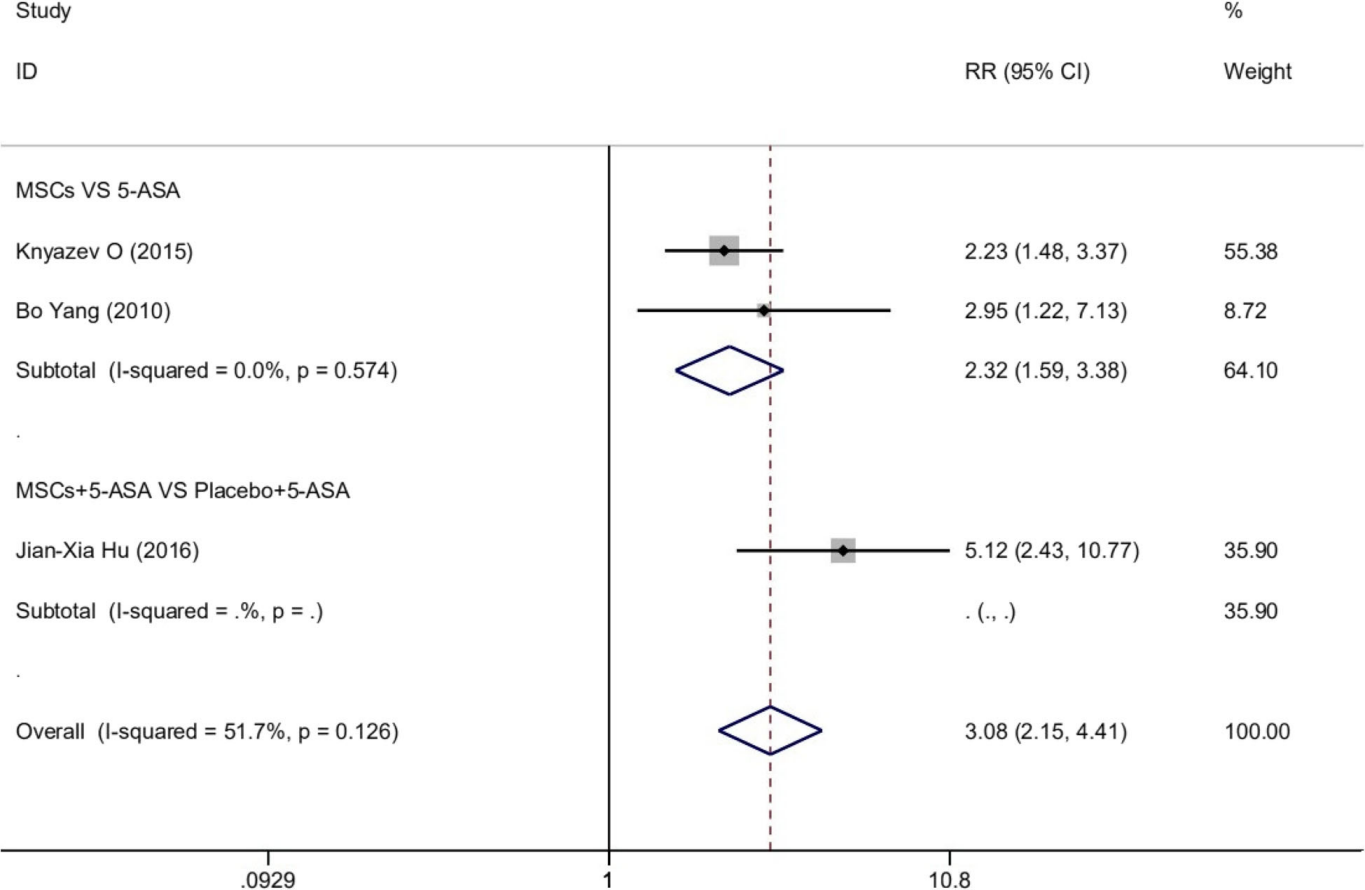
Forest plot of clinical trials with the control group

For 3 articles involved, a total of 99 patients with UC received MSCs, and 96 received conventional treatment. In accordance with varieties of study design, 2 subgroups were defined (MSCs vs 5-ASA and MSCs + 5-ASA vs placebo + 5-ASA). The healing rate in each subgroup was 0.791 and 0.853, respectively. Our analysis showed that MSCs were associated with improved healing rate (HR) as compared with 5-ASA (RR = 2.317, 95% CI 1.591 to 3.375, *p* = 0.000; *I*^2^ = 0%, *p* = 0.574; Fig. 6) and MSCs + 5-ASA were also associated with improved healing rate (HR) as compared with placebo + 5-ASA (RR = 5.118, 95% CI 2.433 to 10.765; Fig. 6).

## Discussion

To the best of our knowledge, this is the first systematic review and meta-analysis to comprehensively summarize the efficiency of MSC in treatment with UC including both animal and clinical trials. Our results have demonstrated that both animal studies and human studies suggest that MSC has more significant therapeutic potential for UC mouse models or patients with UC, compared with conventional therapies.

In animal trials, our static analysis would still be unable to achieve a low heterogeneity on DAI (*I*^2^ = 44.83%) and HS (*I*^2^ = 41.50%) after performing subgroup analysis and linear regression. The possible explanations are presented as follows. Primarily, blindness was not described in all of the 7 studies, which may contribute to heterogeneity and bias. Secondly, UC mouse models were established by the administration of dextran sodium sulfate (DSS) with concentration ranging from 1.5 to 5%. The duration of modeling was from 6 to 30 days. Thus, the differences of modeling could also be associated with heterogeneity. Plus, with regard to the sources of MSCs, five of the seven animal studies used bone marrow MSCs (BM-MSCs) (*n* = 84), one applied umbilical cord MSCs (UC-MSCs) (*n* = 20), and another one adipose MSCs (AD-MSCs) (*n* = 8). There is no denying that more proper studies are required to regulate the modeling and implementation details of the intervention of UC to standardize animal experiments.

A meta-analysis conducted by Fold et al. has a failure to achieve remission in 724 (58.1%) of 1247 patients randomized to receive 5-ASA, and the RR of failure to achieve remission with 5-ASA compared with placebo in active UC was 0.79 (95% CI 0.71 to 0.88). It also seemed that the dose size of 5-ASA revealed no significance on the therapeutic effects (*p* = 0.13) [11]. The outcomes of a meta-analysis from Khan and colleagues suggested a trend for the benefits of azathioprine therapy (healing rate = 69.23%), but it did not reach statistical significance (RR = 0.85; 95% CI 0.71 to 1.01) [49]. Compared with the placebo group, the healing rate of vedolizumab was statistically significant (OR = 2.51, 95% CI 1.18 to 5.48) presented by Vickers and colleagues [50]. Two non-randomized controlled studies included in our study figure out a significant efficacy of BM-MSCs versus 5-ASA control group (0.791, 95% CI 0.696 to 0.887). Despite the absence of control groups in the remaining 4 single-arm studies, the healing rate of MSC therapy (0.787, 95% CI 0.715 to 0.867) was higher than that of the above 5-ASA and azathioprine therapies. Due to the lack of data homogeneity compared with biological agents, more studies are needed for more sufficient evidence.

Apart from the efficiency of MSCs, greater importance should be attached to the safety issue. Of the seven human trials, no life-threatening adverse events were reported. In the study by Liang et al. [44], there were two patients suffering from low fever and insomnia after MSC infusion, respectively. Nevertheless, their symptoms restored quickly within 2 days without any medical intervention. Two kinds of MSCs were applied in our review where 182 patients with UC in six trials were treated with BM-MSCs; 34 patients in one trial accepted UC-MSCs. In consistence with the fact that the bone marrow (BM) has been the major source for the isolation of MSCs, but its invasive donation procedure and the reduction in life span of MSCs along with differentiation potential with growing age may cause injury [51, 52]. Compared with BM, although the successful separation rate of umbilical cord is relatively lower (100% vs 63%), it brings benefits in a less invasive method of being obtained, higher proliferation capacity, and lower colony frequency (*p* < 0.001) [53]. Findings from Shi et al. showed that the clinical application of MSCs derived from UC and adipose tissue has been increasing more than 30% as an alternative source in the past 10 years [25]. Taken together, future clinical applications should not merely be grounded in differentiation capacity, but also on the safety of the stem cells.

In terms of the administration routes of MSCs, which might also contribute to the tremendously various outcomes of MSC treatment, our results illustrated that both the delivery of intravenous injections and submucosal injection by endoscopy could be conducive to the healing and recurrence of UC [18]. It is also reported that submucosal endoscopic injection using AD-MSCs could ameliorate TNBS-induced colitis, especially stenosis in rats [54]. Meanwhile, we have noticed from Nam and Cao’s studies that compared with using entire mouse BM-MSCs, application of extracellular vesicles (EVs) extracted from mouse BM-MSCs was more efficient in improving colon length [38, 48]. Since no trial has been implemented to compare manners of delivery, it remains unclear whether injected MSCs must migrate to sites of inflammation or whether they can exert their therapeutic effects in a systemic way. Lightner et al. [55] reported that the healing rates were higher when MSCs were combined with fibrin glue or a Gore Bio-A Fistula Plug compared with direct injection (71% and 83% versus 50%). It seemed that intravenous, intraperitoneal and submucosal endoscopic injections are all feasible manners to put up significative outcomes in MSCs-therapy. Unfortunately, we were not able to determine which administration routes would occupy predominance due to the low quantity and quality of included literature. In consequence, more studies should be carried out to draw conclusions concerning which method is more reliable and effective.

Despite it is not known the precise mechanisms of UC, recent studies indicated both innate and adaptive immunity play a part in disease pathogenesis [56]. For instance, interleukin-5 (IL-5) produced by Th2-polarized T cells in colonic lamina propria cells, as well as IL-13 came from nonclassical natural killer T cells [57], were found to contribute to epithelial cytotoxicity and barrier dysfunction in UC patients. Meanwhile, the activation of neutrophils and dendritic cells, along with the expression of Toll-like receptors 2 (TLR2) and TLR4, was proved to be accumulated in colonic tissue [58–60]. Legaki et al. modified the expression of cytokines in the UC mouse model using extracellular matrix of cultured MSCs, which successfully reduced intestinal inflammation at pathological level [61]. MSCs might be able to exert protective functions by supporting colonic epithelial cells’ and mucous barriers’ survival and regeneration through the production of growth factors, exosomes, cytokines, and metabolites [62, 63]. They may also serve as the function of immunosuppression which could prevent the activation of effector T cells and promote the formation of regulatory T (Treg) cells [64–66]. In the past 2 years, Park et al. and Yousefi-Ahmadipour et al. have suggested that ASCs have the ability to reduce numbers of inflammatory M1 macrophages and induce differentiation of anti-inflammatory M2 macrophages to alleviate the symptoms of UC [67, 68]. In the future, it is imperative to carry out more research on molecular mechanisms to elaborate the specific association between MSCs and UC.

Our study has certain limitations which are worthy of consideration. Primarily, parts of the enrolled studies are small-sized with low methodological quality. Plus, studies were not extensive enough owing to insufficient location sources. Additionally, we could not assess publication bias. Finally, no histopathologic or other direct indicators are evaluated to estimate the role of MSCs (such as endoscope and MRI) in human studies.

## Conclusion

In conclusion, our results provide a systematic summary on efficacy of MSCs for the treatment of UC. Although MSCs appear to be potentially safe and effective in large numbers of animal and clinical trials, further randomized controlled clinical studies with high quality are needed to offer more powerful medical evidence.

## Additional files

Additional file 1: Retrieval strategy. (DOCX 15 kb)

Additional file 2: Source of heterogeneity (Table S1, Table S2, Table S3). (DOCX 123 kb)

## Data Availability

Availability of data and materials can be assessed both in the Material and methods section and the Results section.
