# Supplementary material for: Mesenchymal stem cells for the treatment of ulcerative colitis: a systematic review and meta-analysis of experimental and clinical studies
Source: Stem Cell Res Ther. 2019 Aug 23;10:266. doi: 10.1186/s13287-019-1336-4 (PMC6708175; doi:10.1186/s13287-019-1336-4)
Supplement: Supplementary file 1 — Retrieval strategy. (DOCX 15 kb) [file 13287_2019_1336_MOESM1_ESM.docx]

**Retrieval strategy**

**PUBMED: 56 records**

#1: ((Ulcerative Colitis[Title/Abstract]) OR Idiopathic Proctocolitis[Title/Abstract]) OR Colitis Gravis[Title/Abstract]

#2: (((mesenchymal stem cells[Title/Abstract]) OR Bone Marrow Stromal Cells[Title/Abstract]) OR Mesenchymal Progenitor Cells[Title/Abstract]) OR Mesenchymal Stromal Cells[Title/Abstract]

#3: #1 AND #2

**EMBASE: 148 records**

#1: (Colitis, Ulcerative):ti,ab,kw OR (Idiopathic Proctocolitis):ti,ab,kw OR (Ulcerative Colitis):ti,ab,kw OR (Colitis Gravis):ti,ab,kw

#2: (Mesenchymal Stem Cells):ti,ab,kw OR (Bone Marrow Stromal Cells):ti,ab,kw OR (Mesenchymal Progenitor Cells):ti,ab,kw OR (Mesenchymal Stromal Cells):ti,ab,kw

#3: #1 AND #2

**Cochrane Library: 5 records**

#1: (Colitis, Ulcerative):ti,ab,kw OR (Idiopathic Proctocolitis):ti,ab,kw OR (Ulcerative Colitis):ti,ab,kw OR (Colitis Gravis):ti,ab,kw

#2: (Mesenchymal Stem Cells):ti,ab,kw OR (Bone Marrow Stromal Cells):ti,ab,kw OR (Mesenchymal Progenitor Cells):ti,ab,kw OR (Mesenchymal Stromal Cells):ti,ab,kw

#3: #1 AND #2

**Web of Science: 204 records**

#1: TS=('Mesenchymal Stem Cells' OR 'Bone Marrow Stromal Cells' OR 'Mesenchymal Progenitor Cells' OR 'Mesenchymal Stromal Cells')

#2:TS=('Colitis, Ulcerative' OR 'Idiopathic Proctocolitis' OR 'Ulcerative Colitis' OR 'Colitis Gravis')

#3: #1 AND #2

**China National Knowledge Infrastructure (CNKI): 37 records**

#1: (SU=' Mesenchymal Stem Cells ' OR SU=' Bone Marrow Stromal Cells ' OR SU=' Mesenchymal Progenitor Cells ' OR SU=' Mesenchymal Stromal Cells ')

#2: (SU=' Colitis, Ulcerative ' OR SU=' Idiopathic Proctocolitis ' OR SU=' Ulcerative Colitis ' OR SU=' Colitis Gravis ')

#3: #1 AND #2
