# Supplementary figures and images for: Mesenchymal stem cells for the treatment of ulcerative colitis: a systematic review and meta-analysis of experimental and clinical studies
Source: Stem Cell Res Ther. 2019 Aug 23;10:266. doi: 10.1186/s13287-019-1336-4 (PMC6708175; doi:10.1186/s13287-019-1336-4)

**Source of heterogeneity**

**Table S1**


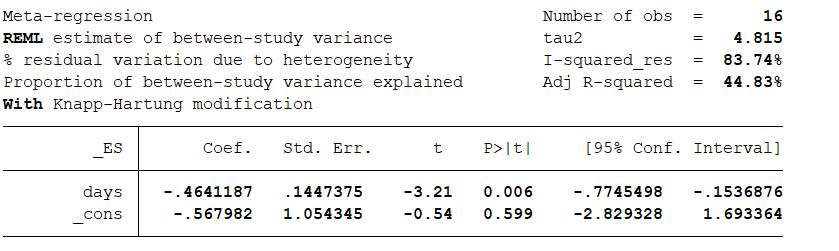


**Table S2**


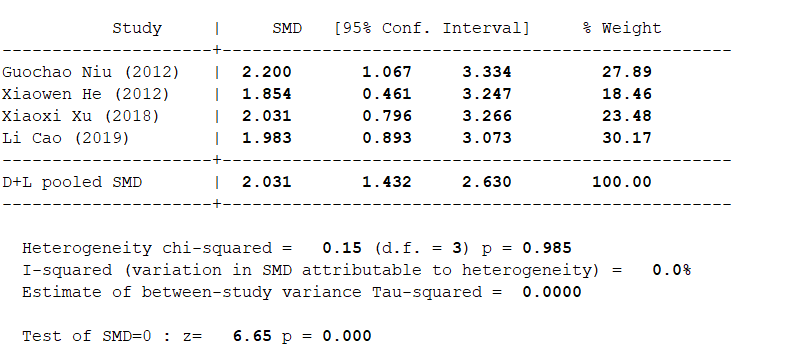


**Table S3**


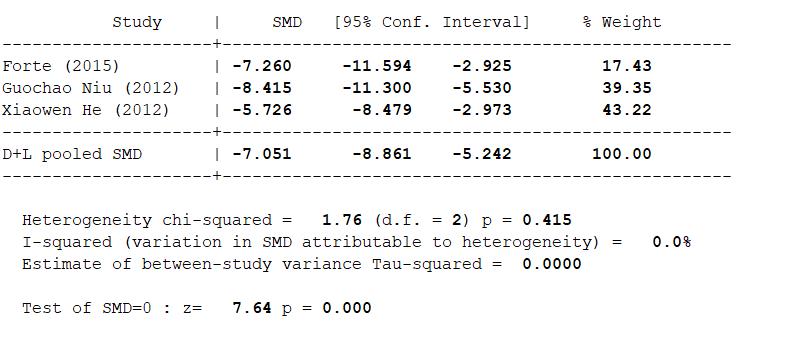

Supplement: Supplementary file 2 — Source of heterogeneity (Table S1, Table S2, Table S3). (DOCX 123 kb) [file 13287_2019_1336_MOESM2_ESM.docx]
